# Smokeless Tobacco Cessation Support in Dental Hospitals in Pakistan: Dentists and Dental Patients’ Perspectives on Current Practices, Support Needed, and Opportunities Available

**DOI:** 10.1093/ntr/ntad125

**Published:** 2023-07-25

**Authors:** Shaista Rasool, Fiona Dobbie, Fayaz Ahmad, Zohaib Khan, Richard Holliday, Linda Bauld

**Affiliations:** Usher Institute, University of Edinburgh, Edinburgh, United Kingdom; Institute of Public Health and Social Sciences, Khyber Medical University, Pakistan; Usher Institute, University of Edinburgh, Edinburgh, United Kingdom; Institute of Public Health and Social Sciences, Khyber Medical University, Pakistan; Institute of Public Health and Social Sciences, Khyber Medical University, Pakistan; School of Dental Sciences, Faculty of Medical Sciences, Newcastle University, United Kingdom; Usher Institute, University of Edinburgh, Edinburgh, United Kingdom

## Abstract

**Introduction:**

Despite evidence on the effectiveness of tobacco cessation interventions in dental settings, the implementation remains low, especially for smokeless tobacco (ST). The purpose of this study was to develop an understanding of the influences governing the implementation of ST cessation support in dental hospitals.

**Aims and Methods:**

A multicenter qualitative study was conducted at two tertiary-care dental hospitals, in Pakistan. Semi-structured interview guide, guided by the Capability-Opportunity-Motivation-Behavior (COM-B) model, were used to capture the views of dentists (*n* = 12) and dental patients (*n* = 12), regarding ST cessation support in dental hospitals. Framework approach was used to thematically analyze the data.

**Results:**

Screening of ST users in routine dental practice was seldom practiced and the cessation support offered was brief advice. Barriers identified by dentists included: Fear of offending and stereotyping patients; lack of knowledge and skills; lack of privacy; lack of belief in the effectiveness of behavioral support; lack of time and workload pressure; ST use amongst dentists; lack of referral systems and; the absence of a mandatory requirement of offering ST cessation support. Facilitators included: Delivering support through junior dentists and the length of interaction between the dentist and the patient. Naswar was the most common ST product used by dental patients. Patients reported receiving negligible cessation support from any healthcare provider.

**Conclusions:**

A range of influences governing the implementation of ST cessation support in dental hospitals were identified. These findings can inform the implementation of behavioral interventions for ST cessation in dental and other clinical settings, in low and middle-income countries.

**Implications:**

Smokeless tobacco control considerably lags, in comparison to the control of combustible tobacco. This is the first study that qualitatively explores the implementation of ST cessation support in dental settings in Pakistan. Utilizing the “Capability-Opportunity-Motivation-Behavior” model, it provides an in-depth understanding of the inability of dentists in implementing effective behavioral interventions for ST cessation support in routine dental practice. Highlighting the striking discrepancy between the patient’s need for and receptivity towards cessation support and the dentists’ concerns over their patients’ receptivity towards cessation support, it calls for the need for effective implementation strategies to optimize dentist-led tobacco cessation interventions in low-resource settings.

## Introduction

Smokeless tobacco (ST) products are noncombustible tobacco products, categorized as group-1 carcinogens by the International Agency for Research on Cancer.^1,2^ The South Asian region, where more than 85% of the 300 million ST users live, reportedly shares the highest burden of disability-adjusted life years and the greatest risk of oral cancers from ST.^3–5^

The prevalence of ST use in Pakistan is one of the highest in the South Asian region. According to the global youth tobacco survey (GYTS 2013) and the global adult tobacco survey (GATS 2014), the prevalence of ST use among adolescents and adults in Pakistan is 7.3% and 7.7%, respectively.^6,7^ Several subnational surveys have reported higher use amongst adults, ranging from 38.2% to 50%, with use amongst adolescents as high as 25%.^8–10^ Naswar, a highly addictive, potentially carcinogenic product, with a high concentration of tobacco-specific nitrosamines,^11^ is reportedly the most popular choice amongst ST users in Pakistan.^7^

Article 14 of the framework convention for tobacco control (FCTC), requires all parties to “offer help to tobacco users to quit.”^12^ All healthcare providers (HCPs) are recommended to provide tobacco cessation support during routine practice. The wider reach of the healthcare systems to the population serves as a great advantage for integrated tobacco cessation services. Amongst the HCPs, oral healthcare providers are a primary source for the identification of tobacco-induced harm in the oral cavity.^13^ Unfortunately, article 14 remains *“*one of the most under-implemented articles*”* of the FCTC. While progress is slow for all tobacco products, the quit support offered to ST users is even less.^12,14^

Despite being a signatory of the FCTC since 2005,^15^ Pakistan lags behind its neighboring countries in terms of ST control laws and alignment of the existing laws with FCTC guidelines.^4^ Pakistan is yet to develop national guidelines for tobacco cessation. Very limited support is offered to tobacco users in terms of cessation and treatment of tobacco dependence. So far, Pakistan has established three cessation clinics. Pharmaceutical products are available at a few pharmacies and their cost is not covered by the government. There is no national quit line. Tobacco dependence diagnosis and treatment are not integrated into the existing health system. There is neither a training provision for HCPs in tobacco cessation nor any guidelines for incorporating tobacco dependence treatment into the curriculum of health professionals. According to GATS 2014, one in five current ST users (20.4%) was thinking about quitting whereas, 21.4% of the ST users had made a quit attempt. Half of these quit attempts (50.8%) were unassisted. These figures reflect the gap between the need for and the availability of cessation support offered to tobacco users.

In Pakistan, as elsewhere, oral healthcare providers can play a key role in tobacco cessation.^16–18^ Evidence suggests that oral healthcare providers are willing to offer tobacco cessation support; however, it is seldom practiced.^19^ There is a growing body of evidence on the barriers to and facilitators of the implementation of tobacco cessation interventions in routine dental practice.^20–22^ This evidence is, however, largely centered on “smoking cessation” with ST receiving little to no attention. Furthermore, there is a lack of theoretical underpinning in the evidence on the barriers to and facilitators of implementing ST cessation support in dental settings. Theoretical insights into how influences govern behavior change are useful in designing and implementing context-driven interventions.^23^ Likewise, much of the evidence on the effectiveness of ST cessation interventions in dental settings comes from high-income countries (HMICs)^24^ which limits its generalizability to low-middle-income countries, who share the greatest burden of the problem.

While low-middle-income countries must integrate ST cessation in dental settings, it is also important to understand that translating evidence into practice requires careful consideration of the context to help identify the influences that will facilitate or hinder the likelihood of such research uptake.^25^ The current study is therefore, guided by the capability-opportunity-motivation-behavior (COM-B), model to help identify the influences governing dentists’ behavior to routinely offer ST cessation support to their patients. The model recognizes behavior as a product of an interrelated system that involves an individual’s or group’s capability (physical and psychological), opportunity (social and physical), and motivation (reflective and automatic).^26^ “Capability” refers to whether an individual has the knowledge (physical capability) and skills (psychological capability) required for engaging in the target behavior. “Opportunity” in the COM-B model refers to all the physical and social “factors that lie outside the individual” that are required for engaging in the target behavior.^*26*^ The COM-B model defines motivation “as all those brain processes that energize and direct behavior, not just goals and conscious decision-making.”^26^ The study also explores the dental patients’ views on dentist-delivered ST cessation support. This study is part of a larger feasibility study, which aims to assess the feasibility of implementing a structured behavior support intervention for ST cessation within dental hospitals in Pakistan.^27^

## Methods

The detailed methods of the study are published in a protocol paper elsewhere.^27^ Briefly, this was an exploratory qualitative study, involving 24 in-depth interviews with dentists (*n* = 12) and dental patients (*n* = 12).

### Study Type and Settings

A multicenter exploratory qualitative study was conducted at five departments of two tertiary-care dental hospitals, in the Khyber Pakhtunkhwa (KP) province of Pakistan. Since little is known about the current clinical behaviors of dentists or the ST use behavior of dental patients, the COM-B model was used to guide this qualitative inquiry (Supplementary Appendix 1, 2). The model provides a framework for performing a behavioral analysis and it has been extensively used to understand behaviors and aid the development of behavioral interventions.^28^

To ensure the sample diversity that this exploratory qualitative study required, the selected hospitals included both public and private sector teaching hospitals. Dentists working in five specialties of dentistry namely: Prosthodontics; periodontics; orthodontics; operative dentistry; and; maxillofacial surgery and dental patients (who were ST users) visiting the hospitals were eligible to participate in the study.^27^

### Participants and Recruitment

#### Dentists

Twelve dentists were interviewed, recruiting an equal number of dentists (*n* = 6) from each of the two study sites. A purposive sampling technique was employed for the selection of dentists to ensure representation from both sexes (male, *n* = 8 and female, *n* = 4). At both sites, the senior-most dentist(s) from each of the five specialties were recruited. This was based on the premise that these dentists would have accumulated experience, having gone through the whole spectrum of job responsibilities across the dental care system in tertiary-care hospitals, ranging from clinical care provision to supervising clinical residents and leading a whole specialty department. Thus, providing in-depth insights and diversity. Dentists were invited face-to-face and an appointment was scheduled with them after seeking their informed consent.

#### Dental Patients

Twelve patients (who were ST users) were purposively selected from the general, outpatient department (OPD).^27^ Dental patients visiting the OPD were invited after their tobacco use history had been documented by the OPD consultant. The interview appointment was scheduled with them after seeking their informed consent.

### Interviews

#### Dentists

All interviews were conducted in person by SR at the offices of the interviewees. The interview duration ranged from 26 to 53 minutes. The topic guide covered the dentists’ capability, the opportunities available to them and the factors influencing their motivation, in relation to offering ST cessation support. Additionally, the guide also included questions exploring the current clinical practices of dentists concerning ST cessation support.

#### Dental Patients

All interviews were conducted in person by SR at the offices of dentists at the hospital. Apart from two interviews where patients were accompanied by relatives, all the other interviews included the interviewer and interviewee only. The patients’ interviews ranged from 19 to 52 minutes. The topic guide explored a wide range of topics including the patients’ ST use behavior (initiation, continued use, and cessation attempts), factors influencing those behaviors and their views on ST cessation support in dental care.

## Analysis

All interviews were conducted in local languages (Pushto or Urdu) except for an interview with one dentist, which was conducted in English. The interviews were audio recorded and transcribed verbatim by SR. Field notes were taken by SR after each interview to provide context for the analysis, later. The transcripts were then analyzed thematically using the framework analysis approach. This began with familiarization with the transcripts to create a coding frame (SR, FD, and FA). Coding was inductive and while the topic guides were guided by the COM-B model, in that, the questions related to the behavior of dentists and patients were categorized under the three domains of the model, these predefined categories of the COM-B model were however, not applied to the data at the time of coding the transcripts. Data saturation were discussed and confirmed before finalizing the coding frame, after which the transcripts were indexed and sorted by labeling sections of data against the relevant code. This was followed by summarizing the data, which are the final step of data management using the framework method. A summary of each coded section of the transcript was written for each case and was displayed under the relevant sub-theme in a set of matrices using MS Excel spreadsheets, allowing for an across and between-case comparison of the themes. Data abstraction and interpretation followed ordering and summarizing data, which lead to the identification of several themes, discussed in the results section. The study adhered to the consolidated criteria for reporting qualitative research (COREQ). Results are presented using the domains from the COM-B model with illustrative quotes from dentists’ and patients’ interviews provided in Tables 1 and 2, respectively.

**Table 1.** Influences Governing Dentists’ Clinical Behavior of Offering ST Cessation Support During Routine Dental Practice

Theme	Sub-theme	Sample quote
1. Clinical behaviors	1a. Taking history of smokeless tobacco use 1b. Offering cessation advice	*1. “*We rarely ask about naswar from our patients, we don’t ask, until and unless they present with very questionable oral hygiene.*”(Male Dentist ID9)* *2.“*We teach our trainees about taking ST use history and it’s always there in the history charts but if you are talking about its implementation, it’s not followed for common procedures.” *(Female, Dentist ID11)* *3. “*The ones(patients) above 60/70 years, say we have spent our life using naswar and we also say, let them have it. That’s all the joy left with them.” *(Male, Dentist ID2)*
2. Influences governing dentists’ clinical behavior	2a. Capability 2b. Opportunity 2c. Motivation	*4. “*I don’t remember it (5As approach) exactly at the moment though. We just learn for exams. I don’t remember the exact protocol. Because we don’t practice it.” *(Male, Dentist ID1)* *5. “*If I stop you from drinking tea, will you stop? You know more than me that tea has the same nicotine as naswar[….] Excess of everything is bad, it’s definitely not OK to use naswar because there is no stopping, but we can place a limit.” *(Male, Dentist ID2)* *6. “*Taking history from females is very difficult. They don’t disclose easily. They are always reluctant and so are we. And rest, the people who are above 40 years they disclose very easily. We don’t face any difficulty with them.” *(Male, Dentist ID10)* *7. “*A few might be using naswar for recreation but a few might be stressed, if you listen to their stories, you don’t have enough to offer. We aren’t professionally trained to treat depression and anxiety.” *(Male, Dentist ID2)* *8. “*I have a lot of things to worry about. I have to run this department, supervise trainees, my research work, and many other things, why should I be bothered whether my patient uses tobacco or not?” *(Male, Dentist ID1)* *9. “*Counseling should not be done in the middle of a clinical department with all chairs (dental units) occupied around, because there are distractions.” *(Male, dentist ID9)* *10*. “Time (for offering ST cessation support) isn’t an issue in our ward, as we (the ones who are experts), take 45 minutes on one patient. House officers and the rest have up-to two hours to spend on one patient. So time barrier doesn’t apply here.” *(Female, Dentist ID6)* *11*. “Senior most dentists here are preoccupied in multiple tasks, the junior staff is only involved in clinical work, so they can effectively cover up for this, they will be able to counsel at least one patient per day.” *(Male, Dentist ID 4)* *12*. “Another thing is that the patient will start doubting us and will think that I am here for a dental treatment. Why is he talking about tobacco with me?” *(Male, Dentist ID4).* *13*. “But there are so many restrictions here. We can’t ask female patients if they use naswar. I am in no mood to get beaten by them.” *(Male, Dentist ID1).* *14*. “We don’t directly ask about naswar, they feel embarrassed disclosing it. We can ask about cigarette smoking, but about naswar, we can’t ask anyone. Patients feel uneasy and so do we.”(*Female, Dentist ID6)* 15. “Naswar use amongst dentists is very common, we would do oral cancer surgery during the day and my friend (dentist) would use naswar at night.” (Male, Dentist ID8) *16*. “It is our duty and responsibility to educate the patient. Now if we can tell the patients that you shouldn’t use a lot sweets, that you should brush your teeth before sleeping, that you have to brush your teeth after breakfast, then why not this (ST use).” *(Female, Dentist ID6)* 17. ‘Dentists aren’t concerned if the patient quits or not. Of course we know the risks very well. But it all boils down to “what have I got to do with my patient using tobacco? Why should I be bothered if he smokes or uses naswar?” (Male, dentist ID1) *18*. “I don’t have time to sit with patients and counsel them to quit. Do you think I have less things to worry about that I sit with patients and talk to them about tobacco.” *(Male, Dentist ID1)* *19*. “Maybe he quits later, but you see, I don’t get the results as a clinician, my patients aren’t responding (giving up naswar) like that.” *(Female, Dentist ID5)*

**Table 2.** Influences Governing Dental Patients’ ST Use Behavior

Theme	*Sub-theme*	*Sample quote*
Influences governing ST use behavior of dental patients	1a. Capability 1b. Opportunity 1c. Motivation	*1*. “I honestly didn’t know what naswar is made of. I didn’t know it has slaked lime and it has these side effects.” *(Male, Patient ID5)* *2*. “I will bring a woman to you who uses naswar. You can ask her about the risk to pregnancy from naswar use. She has many children. All her children are fine.” *(Female, Patient ID8)* *3*. “All these health risks from naswar use, I don’t believe in them. I don’t see any negative effect.” *(Male, Patient ID2)* *4.* “My uncles died at a very young age due to a rare familial disease (in which a pustule develops into cancer). They didn’t use naswar, but my father used naswar, so when he got this pustule his symptoms weren’t that severe. My father told me that naswar provides protection from this disease.” *(Male, Patient ID7)* *5.* “I have tried to quit it, but it doesn’t work, I feel like I’ve lost something, as if somethings missing. I get headaches.” *(Male, Patient ID6)* *6*. “I am helpless, something or the other comes up. Some incident, something happens and that reminds me of naswar.” *(Female, Patient ID10)* *7*. “No one (HCP) has ever advised me or counseled me about this. I know that I need support, I need counseling, I really want to quit.” *(Male, Patient ID6)* *8*. “I was five, my mother had passed away, a woman approached me and told me to use naswar, and she said it will calm me down, that it will calm my nerves.” *(Female, Patient ID10)* *9*. “Once I had naswar in my mouth and some guests came. They were very respectable, white collar people. I couldn’t spit it out in front of them, I felt ashamed of myself. I couldn’t leave the place. That’s when I realised how disgusting and shameful this habit is.” *(Male, Patient ID6)* *10*. “I didn’t use it (naswar) for two months, twice. Then I thought to myself, why have I even quit? It wasn’t causing me any harm. Except that it’s not socially acceptable, so I said to myself, fine, I won’t use it where it’s not acceptable.” *(Male Patient ID2)* *11*. “I know I want to quit all the time. If the place is dirty, it’s OK, but if it’s a clean place like this place (dentist’s office) and I spit, I feel so bad, I hate myself for it.” *(Male, Patient ID5)* 12. “This (naswar) is chickens” poop that I put in my mouth, but what I can do now, I’m addicted. It’s a habit, nothing else.’ (Male, Patient ID1)

## Results

### Participants Characteristics

The interviews were conducted from September 22 to December 13, 2021. Table 3 summarizes the participants’ characteristics.

**Table 3.** Demographic Characteristics of Study Participants

Demographics	Patients	Dentists
Age(years)(mean ± SD, min, max)	44.4	43.3years
	SD = 12.5	SD = 8.8
	Min = 27	Minx = 35
	Max = 60	Max = 63
Sex (*n*/%)		
Female	3(25%)	4(33%)
Male	9(75%)	8(67%)
Education		
No education	3(25%)	
Grade1–4	1(8%)	
Primary	2(17%)	
Middle	1(8%)	
Secondary	—	
Higher Secondary	1(8%)	
Bachelors	2(17%)	
Masters	1(8%)	
Occupation		
Retired	1	
Unemployed	3	
Street vendor	1	
Driver	5	
Marketing job	1	
Self-employed	1	

### Dentists’ Interviews

Two main themes that emerged from the dentists’ interviews were, “clinical behaviors” related to ST cessation support and “perceived influences” governing the dentists’ behavior of delivering ST cessation support. All dentist interview quotes referenced in this section are presented in Table 1.

#### Theme 1: Clinical Behaviors

The two subthemes that emerged under “clinical behaviors” were “taking a history of ST” (1b) and “offering cessation advice” (1c) (Table 1). Regarding history taking, the responses varied between the specialties. For instance, dentists working in the maxillofacial surgery department reported having no requirement for taking ST use history, except, when patients presented with compromised oral hygiene, suspicious lesions, or ulcers (Quote 1). Whereas those working in the periodontology, prosthodontics, and oral medicine departments, reported having a mandatory requirement for ST use history from all patients, however the same was not practiced for all patients (quote 2). In most cases, the nature of the ST cessation advice was: brief, not related to the specialty of the dentist; tailored to the patient’s socioeconomic profile and; guided by the dentists’ beliefs about naswar use and the consequences of the advice. Specific advice offered, included one or a combination of the following: Creating awareness about the associated health risks; instilling fear (by emphasizing oral cancers); advising the patients to quit or reduce the use of ST and; educating them about the significance of periodontal health for the success of dental treatment. Most dentists did not offer any advice to elderly patients (quote 3). Advising patients who were not ready to quit, to alternate between the different sides of the mouth for the placement of naswar, was also reported.

#### Theme 2: Influences Governing Dentists’ Clinical Behavior

Dentists identified several influences that hindered or facilitated their clinical behavior related to the implementation of ST cessation support in routine practice. These factors have been categorized according to the three domains of the COM-B model into: Capability (2a); opportunity (2b) and; motivation (2c) (Table 1). All of the six sub-domains (*physical and psychological capability, physical and social opportunity, reflective, and automatic motivation*) were discussed.

##### Capability.

Capability here refers to the dentists’ psychological and physical capacity to engage in “ST cessation support in routine dental practice.” With regards to *psychological capability (knowledge),* while dentists were well aware of the use of naswar amongst their patients, there was an overall lack of familiarity with tobacco cessation guidelines. Those who had some awareness about the guidelines were all young dentists (below 40 years), who had learned about the guidelines to pass specialist exams (quote 4). There was good knowledge about the oral health risks associated with naswar use, however, exceptions existed, as one dentist, who did not seem convinced about the risks associated with naswar use, believed that tea contained as much nicotine as naswar (quote 5).

Dentists felt confident in their counseling skills (*physical capability*), they however, expressed an inability to initiate a discussion and ask about ST use from their patients, due to fear of offending the patients (quote 6). They also expressed an inability to counsel patients who used ST to manage mental health disorders (quote 7).

##### Opportunity.

The opportunity here refers to all factors that lie outside the dentist, which might facilitate or appear as barriers to delivering cessation support. Time, space, workload, human resources, and lack of policy were the factors highlighted that fell under the subdomain of “physical opportunity.” *Time available to counsel patients in routine ranged from a minimum of 1–2 minutes to a maximum of 15 minutes, whereas, some had no time for interacting with patients, due to their additional administrative roles (quote 8).*

The only space available to counsel patients in a dental hospital was at the chairside, in the clinic. This, the dentists believed, was the least favorable place for offering cessation support to patients, especially because of the noise and lack of privacy (quote 9). While some dentists expressed concerns over patients’ time, they also believed that implementing behavioral support was practical, owing to the lengthy dental visits (quote 10). Leveraging support from junior and midlevel dentists with no administrative responsibilities, was a common facilitator as many viewed these dentists as an untapped resource and a potential facilitator (quote 11).

The common barriers, discussed under the subdomain of “social opportunity” included; dentist-patient relations and sociocultural norms. Dentists feared that asking about ST use or offering advice might be met with suspicion and adversely influence their relationship with the patients (quote 12). Regarding sociocultural norms, dentists believed that while naswar use was common in patients from rural and uneducated backgrounds, its use was declining and was looked down upon with growing education. The dentists’ inability in asking their patients about naswar use, partly stemmed from their fear of stereotyping patients due to the social disapproval associated with naswar use in the educated segments. This disapproval was reportedly more in the case of females, as “well-mannered” females were not expected to use any form of tobacco (quote 13). While it is not socially acceptable for females to use any type of tobacco, for males, the social disapproval amongst the educated is less with cigarette smoking, which is why dentists felt confident in taking the history of smoking but found it challenging to take ST use history (quote 14).

##### Motivation.

The influences that seemed to drive the dentists’ motivation included: Role identity (professional and social; ST use amongst dentists *(automatic motivation)* (quote 15*);* beliefs about the effectiveness of dentists’ advice and; lack of incentives *(reflective motivation).* Overall, dentists appeared cognizant of their general responsibility towards creating awareness among patients about any habit that may adversely influence their health (quote 16). However, they also argued that offering cessation support meant pushing the boundaries of their role as a dentist/clinician/senior faculty (quote 1 and, 18). It was widely agreed that the inclusion of ST cessation support in routine dental practice will be a valuable addition; however, it was viewed as a task best suited to; a specific department, cessation clinic, support staff, or junior dentists. The dentists’ lack of belief in the effectiveness of their advice was also an important barrier, hindering the implementation of ST cessation support (quote 19).

### Patients’ Interviews

#### Theme 1: Influences Governing ST Use Behavior of Dental Patients

“Naswar” was the only ST product used by the patients, except for one patient who reported occasional use of “khaini” (in addition to regular use of naswar) and another who had switched from “paan” to “naswar.” The influences governing ST use were categorized according to the three domains of the COM-B model: Capability (1a); opportunity (1b) and; motivation (1c) (Table 2). All six sub-domains of the COM-B model appeared to influence the ST use behavior of dental patients. All patient interview quotes referenced in this section are presented in Table 2.

##### Capability.

With regards to *psychological capability*, male patients had good awareness about the ingredients of naswar, whereas females had little to no knowledge. However, exceptions existed, as a few male patients had little knowledge about the ingredients of naswar (Quote 1). Their views regarding perceived health benefits and harms of naswar were mixed, at best. While patients had experienced oral health harms from naswar use, they had little knowledge to believe in the risk of oral cancer, cardiovascular diseases, and risk to pregnancy from naswar use (quotes 2 and 3).

Stress management, was reportedly the most common perceived health benefit, followed by relief from toothache and maintenance of regular bowel movements. Some also believed that naswar use made them feel energized. Contradicting their belief of naswar being beneficial for maintaining regular bowel movements, many reportedly experienced problems with digestion from naswar use. Interestingly, two patients, believed that naswar use weakened their eyesight, attributing it to loss of saliva, due to too much spitting (from naswar use). One patient believed in the protective effect of naswar against a rare familial disease (quote 4).

While most participants had remained abstinent for months in the past, they overwhelmingly reported an inability to break the habit of naswar *(physical capability).* Giving in to strong urges, inability to cope with withdrawal symptoms, company of users, stress, resorting to other forms of tobacco, lack of cessation support, or simply lack of determination had persistently rendered the strategies they used to remain abstinent ineffective (quote 5 and 6).

##### Opportunity.

Opportunity influences included a lack of cessation support (physical opportunity) and social norms (social opportunity). While all patients (except one) had attempted a cessation attempt, none had been advised by their healthcare provider in this regard *(physical opportunity)* (quote 7*).* The patients’ initiation and continued use of ST, their past quit attempts and their current desire to quit were strongly governed by social influences (social opportunity). For instance, the uptake of ST in all cases was upon the suggestion of a friend or relative (quote 8). While social influences were critical in the patients’ initiation of ST use, the social disapproval associated with naswar use; however, led patients to conceal or avoid its use in the presence of others (quote 9 and 10).

##### Motivation.

Patients overwhelmingly desired to quit ST use. This desire stemmed from their beliefs and emotions about the social consequences of its use *(reflective motivation*) (quote 11). The general discount for long-term health outcomes combined with the perceived health benefits and the urge to place something in the mouth had made the use of ST a habit (*automatic motivation)*, which they found challenging to quit (quote 12).

#### Theme 2: Patients’ Views on ST Cessation Support in Dental Hospitals

Patients’ views on accessing cessation support during routine dental care were influenced by a range of factors. They overwhelmingly expressed the need for cessation support from dentists, considering them well-placed to provide counseling-based, ST cessation support. Overall, patients seemed willing to take out time during their dental visits for ST cessation support. This willingness was due to the motivation to quit for some, for others, it was driven by the patient's feeling of being excluded from treatment plans and the expectation to follow the plan of their dentists, as remarked by one patient,

*“I will have to give it (dentist-delivered cessation support) time, what can I say if the doctor wants to talk, what I can say?” (Female, Patient ID12)*

While patients found it practical to take out time for cessation support during dental visits, they also mentioned that it will vary from patient to patient, depending upon the patients’ jobs and other commitments. Other concerns raised were a worry about lost wages, attendants’ time, and permission to be away from their job. However, due to the long waiting hours at dental hospitals and the investigations required, they felt that spending a few more minutes on cessation support was not a problem.

## Discussion

This is the first qualitative study conducted in Pakistan that explored the implementation of ST cessation support via dentists from both the patients’ and the dentists’ perspectives. It adds to the evidence from previous research regarding the influences governing the implementation of ST cessation support in dental hospitals. Although dentists acknowledged their role in tobacco cessation, they reported an inability to deliver it for a range of reasons. The perceived barriers included a range of influences that have been reported in the literature: Lack of knowledge and skills; lack of time; lack of belief in the effectiveness of dentists’ advice; lack of supporting resources^21,29^; lack of referral systems^30^; use of ST amongst dentists^21,31^ and perceived patients’ resistance.^19,32,33^ Junior dentists exhibited greater awareness about tobacco cessation guidelines, in line with another study conducted in England.^29^ This can be explained by the recent addition of the requirement for continued medical education or more emphasis on history taking during specialist exams in recent times. The barriers related to social and cultural restrictions, which emerged as one of the most striking factors, have been reported in the literature about other topics, such as alcohol screening and support^34^ or in the implementation of support concerning HPV-related practices.^35^ The same has, however, seldom been reported about ST screening and support. A possible explanation could be that these barriers to ST cessation are specific to certain cultures, where limited relevant research has taken place, such as in Pakistan.

Dentists’ involvement in tobacco cessation has improved in recent years, but it is still limited in Pakistan.^36–38^ This may explain the findings of this study, which differ from recent research in oral health settings in other countries.^39,40^ For instance, asking about tobacco use from all dental patients is a common practice in many countries, including in some South Asian countries like India.^39,41^ Likewise, the practice of taking a history of smoking from all patients, was reportedly as high as 72.8% in Karachi, Pakistan.^38^ A possible explanation for this discrepancy could be that much of the existing evidence on dentists’ practices in tobacco cessation is focused on “smoking cessation” with ST receiving little attention. Research suggests a strong link between contextual factors and health behaviors, with many South Asian studies reporting greater use of ST amongst individuals with low education and income.^42,43^ Individuals belonging to socioeconomically disadvantaged segments have greater exposure to stresses, because of their environment and fewer resources to cope with these stresses.^44^ Such individuals are less likely to quit because of these factors, which in turn can lead to greater dependence.^45,46^ These influences combined with the cultural barriers specific to ST use, scarcity of referral centres,^27^ and unavailability of free nicotine replacement therapy in Pakistan serve as potential barriers translating into the dentists’ inability to identify ST users and their reluctance to offer cessation advice to their patients. Another reason that explains the difference in the history-taking practices of dentists, is that many studies and all the evidence from Pakistan on this topic is quantitative in nature. Qualitative and quantitative findings can sometimes diverge.^47^ This is supported by the initial affirmative response of the dentists in the current study when asked about taking ST use history from patients. However, when probed further dentists reported that, while history taking is mandatory and part of the standard practice that they teach their students, they only take the history of ST use when needed. An interesting finding from this study was the striking discrepancy in a specific notion between the patients and dentists. While on the one hand, patients overwhelmingly expressed the desire to quit and exhibited positive attitudes towards dentists delivering support, the dentists on the other hand believed that ST users rarely want to quit and that offering support might offend patients, supporting the findings on patients’ perspective about tobacco cessation (in dental settings) in the literature.^48^

A key limitation of the study is that only the heads of departments or senior dentists were recruited, instead of those of varying seniority. The findings of this study, therefore, do not represent the views of junior and midlevel dentists, whom the senior dentists viewed as an “untapped dental manpower” for implementing ST cessation support. We recruited a diverse group of dental patients, including males and females across different age groups, who used naswar and offered a diversity of views. A key strength was the recruitment of three Pakistani female ST users from Peshawar, KP in this study. There have been no representations of female ST users from KP in previous literature.^49^ Second, we recruited from a tertiary dental hospital, which caters to both urban and rural populations in KP and beyond, thereby, attaining a diverse sample having representation from the province and beyond.

## Conclusion

Dentists are aware of their role in ST cessation but face several barriers in implementing tobacco cessation and actively contributing to supporting their patients to quit. All previous cessation attempts by dental patients were unassisted, with patients reportedly receiving no cessation advice from dentists. Converging findings from the two subgroups builds on the existing evidence and strengthens the need for dentists’ involvement in ST cessation in low-resource settings.

## Supplementary Material

A Contributorship Form detailing each author’s specific involvement with this content, as well as any supplementary data, are available online at https://academic.oup.com/ntr.

ntad125_suppl_Supplementary_Appendix_S1

ntad125_suppl_Supplementary_Appendix_S2

ntad125_suppl_Supplementary_Appendix_S3

ntad125_suppl_Supplementary_Appendix_S4

ntad125_suppl_Supplementary_Appendix_S5

## Data Availability

The data generated and analyzed during the study are available from the corresponding author upon reasonable request.
