# Supplementary material for: Smokeless Tobacco Cessation Support in Dental Hospitals in Pakistan: Dentists and Dental Patients’ Perspectives on Current Practices, Support Needed, and Opportunities Available
Source: Nicotine Tob Res. 2023 Jul 25;26(1):63–71. doi: 10.1093/ntr/ntad125 (PMC10734380; doi:10.1093/ntr/ntad125)
Supplement: ntad125_suppl_Supplementary_Appendix_S1 [file ntad125_suppl_supplementary_appendix_s1.docx]

**Appendix 1: Topic Guide (Interviews with Dentists)**

Welcome and thank you for making time for this interview.

Purpose and Format of the Interview:

We are conducting a study assessing the feasibility of providing quit support (via dentists) for dental patients who use smokeless tobacco(ST). In this regard we would like to hear your views on implementation of behaviour support interventions for ST cessation within a dental setting. We would like to ask you some questions about ST cessation and dental services offered at your hospital.

We are appreciative of your giving us time and would request as thorough responses as possible.

**Challenges to delivering dental treatments to patients at this hospital?**

1. (Tell me about the dental treatments that you provide to patients at this hospital?)
2. What are the major challenges of delivering these treatments to patients at this hospital?
3. What are the major facilitators of delivering these treatments to patients at this hospital?

**Patients and smokeless tobacco**

1. How common do you think it is for your patients to use smokeless tobacco?
2. Which type of ST product do they use?
3. Do you think the use is different across different age group?
4. What about gender?
5. In your opinion, what are the possible reasons patients might use ST?
6. Is tobacco currently part of any routine recording, reporting, monitoring or supervision mechanisms? If not, do you think this would be useful and feasible addition?
7. Do you ever ask patients about their ST use?
8. How easy do you think they find it to talk to you about using ST?
9. Does this differ between men and women?
10. How?
11. What do you currently do if one of your patients says they use ST? {Probe into process of engagement with patient, steps taken, etc.} Do you offer any advice? any support?
12. How do you think dental patients would respond to being offered support to quit ST use?
13. What misconceptions to you think patients have about tobacco (all forms), and any links with their overall health and particularly oral health?

**Delivery of Smokeless Tobacco Cessation Support**

1. What are your thoughts about behavioural interventions for ST cessation?
2. How would you recommend integrating such interventions effectively within the routine working of dentists at the hospital?
3. What factors need to be considered in regard to providing counselling to patients?
4. What do you know about the role of dentists in tobacco cessation (Probe into 5As).
5. Do you practice the 5As approach in routine practice? If yes, how easy is it to deliver it? If no, then why?
6. What do you think the facilitators and barriers are to delivering ST cessation interventions (for instance mention 5As) within this dental hospital and other clinical dental settings? (Probe on availability of resources, training, monitoring, supervision, time availability of health professionals and the layout of facilities, prescribing/discussing the cessation drug with patients)
7. Realistically, how long do you normally have available to counsel your patient to quit tobacco use?
8. What support from the health department/MoH would you/other staff in the facility need in order to provide tobacco cessation to dental patients?
9. Do you feel your current workload would allow you to offer tobacco cessation to your patients?
10. Where would you do the counselling?
11. How confident do you feel offering support to help dental patients to quit ST use? Or delivering 5As if answer to 21 was yes.
12. Do you think you/other dentists/oral health professionals need any additional skills to deliver ST cessation? What is the best way of increasing skills in this area?
13. From your experience as a health professional, do you have any recommendations on how best to build rapport and communicate well with patients?
14. Do you have any recommendations on how best to support illiterate patients to quit ST?
15. Or any differences in how you would deliver this to men or women?
16. Would you want to deliver this any differently?
17. How valuable/important do you think it would be to add ST cessation to routine dental services?
18. What would it take to influence/convince dental professionals to deliver ST cessations
19. How common is it for health professionals to use tobacco (particularly smokeless tobacco)? Tell me about any experiences you have on this or any policies about this in your facility? Do you think this could have any implication in terms of delivering the intervention?
20. Is there anything else you would like to say about how smokeless tobacco cessation interventions should be delivered in facilities like yours?
21. Would you want to get feedback on the key points and clarify anything you are unsure of?

Thank you for your time today.
