# Supplementary material for: Smokeless Tobacco Cessation Support in Dental Hospitals in Pakistan: Dentists and Dental Patients’ Perspectives on Current Practices, Support Needed, and Opportunities Available
Source: Nicotine Tob Res. 2023 Jul 25;26(1):63–71. doi: 10.1093/ntr/ntad125 (PMC10734380; doi:10.1093/ntr/ntad125)
Supplement: ntad125_suppl_Supplementary_Appendix_S2 [file ntad125_suppl_supplementary_appendix_s2.docx]

**Appendix 2: Topic Guide (Interviews with Participants)**

Welcome and thank you for making time for this interview.

Purpose and Format of the Interview:

We are conducting a study assessing the feasibility of providing quit support (via dentists) for dental patients who use smokeless tobacco. In this regard we would like to hear your views on receiving a behavior support intervention for ST cessation within a dental setting. First we would like to ask you some questions about your ST use and if you have tried to quit before.

We are appreciative of your giving us time and would request as thorough responses as possible.

1. What smokeless tobacco (ST products) do you use? Why those products? (you can show examples of products to encourage discussion)
2. Do you remember when you started using (how old were you?)?How long have you been using ST products?
3. How frequently do you use ST products? (per week/per day) When is this (after waking up, after meals, before toilet, when in stress, before bed, while at work etc.)? How much do you use?
4. How soon after you first wake up do you start chewing?
5. Does anyone in your family use ST? What ST products do people you know use?
6. Do you use ST products more when you are with other people?
   1. Who with?
   2. On what type of social occasions?
7. Would you ever decline ST products on those occasions?
8. Are there any health reasons for why you use ST products? – What are they? Tell me about your experience of this.
9. Are there any health reasons for why you should not use ST products? – What are they? Tell me about your experience of this.
10. What is contained in ST products? (ask about the products they mentioned earlier)
11. Where did you learn this?

(Read out the statement and ask participants to say if they agree/disagree and why)

1. ST controls morning sickness
2. ST leads to an under-developed baby
3. ST damages the gums.
4. ST keeps teeth strong and the mouth clean.
5. ST causes mouth infections.
6. ST helps with digestion.
7. ST relieves constipation.
8. ST prevents sleep.
9. ST is addictive and difficult to give up on your own.
10. ST relieves stress.
11. ST gives strength to do physical activities all day.
12. ST makes you look attractive.
13. ST reduces appetite.
14. ST reduces oral pain.
15. ST can cause miscarriages or still births.
16. Has anyone you know ever tried to quit or reduce ST use? What happened?
17. Have you ever tried to quit or reduce ST use? – how many times? When? **(If no, then go to Intervention delivery)**
18. What prompted you to try to quit or reduce ST?
19. What strategies did you use to try to quit/reduce ST?
20. Did you use professional support? If not, why? T
21. What was the outcome? - E.g. quit for a while, reduced ST use and sustained this.
22. Why did that outcome happen? – E.g. Why did you relapse?
23. What would have helped you to sustain quitting?

**Intervention Delivery**

1. Realistically, how long do you normally have available to be counselled by your dentist to quit tobacco use?
2. How often could you come to a meeting with your dentist?
3. What do you think the facilitators are to receiving tobacco cessation support within this dental hospital and other clinical dental settings?
4. What do you think the barriers are to receiving tobacco cessation support within this dental hospital and other clinical dental settings?
5. Is there anything that would help you to attend the counselling session with the dentist?
6. Is there anything that would make it difficult for you to attend the counselling session with the dentists?
7. Is there anything else about ST use, quitting or the cessation intervention that you want to say?
