# Supplementary material for: Smokeless Tobacco Cessation Support in Dental Hospitals in Pakistan: Dentists and Dental Patients’ Perspectives on Current Practices, Support Needed, and Opportunities Available
Source: Nicotine Tob Res. 2023 Jul 25;26(1):63–71. doi: 10.1093/ntr/ntad125 (PMC10734380; doi:10.1093/ntr/ntad125)
Supplement: ntad125_suppl_Supplementary_Appendix_S3 [file ntad125_suppl_supplementary_appendix_s3.docx]

**Appendix 3 Interview procedure and reflexivity (positionality) statement**

Interview procedure

The interviews were conducted between 22^nd^ September to 13^th^ December 2021. The interviews were conducted by the lead author (SR), a female research dentist conducting a PhD. The researcher had appropriate training having completed a Master’s in Public Health (general qualitative research training) and three training workshops (Fieldwork skills for qualitative social research; Data analysis in qualitative social research; Iinterviewing skills and conducting theory domain interviews). Additionally, she had the experience of conducting in-depth interviews with tuberculosis workers in the past for a research project. The researcher also had day-to-day support from experienced qualitative researchers (FD and FA). There was no relationship between the researcher and participants (dental patients) prior to study commencement, however some of the dentists interviewed were known to her as colleagues as she had previously studied and worked at one of the study sites as a dentist. 13 dentists were invited for interview, one dentist declined to participate due to busy schedule. Amongst the patients, all 13 patients invited, agreed to participate. Amongst these one patient was an ex-user. He who was recruited for the interview due to a miscommunication/misunderstanding of his current ST use status. His interview has not been included in the analysis. The interview topic guides were pilot tested prior to conducting the interviews.There were no conflicts of interests and the interview schedule included a statement reassuring participants that there are no right or wrong answers, answers would not influence future care (in case of dental patients). The aims, objectives and methods of the study were explained to the participants and they were given a participant information sheet which outlined the same in detail. No follow-up interviews were conducted and neither was the participants feedback on the findings sought.

Reflexivity (positionality) statement

Reflexivity is a significant element of qualitative research and is the process of reflecting on how the researcher could influence the research conduct and findings. This is particularly important on the topic smokeless tobacco.

For this study I was the main researcher, a 38-year-old Pakistani female from a middle class background. Having earned a bachelor’s degree in dental surgery and master’s in public health I live and work in Pakistan. On the subject matter of smokeless tobacco, I have never used ST but as a dentist I am aware and have experienced first-hand the extensive health harms that its use can cause. Oral cancer is the leading type of cancer amongst males in Pakistan and the use of naswar is very common amongst males. From previous clinical work which involved treating dental patients in Peshawar where the use of ST is common, I could see the oral manifestations from naswar use, however very little was offered by dentists in terms of ST cessation support. My research aspirations therefore are to help increase the implementation of tobacco cessation interventions in low middle income counties like Pakistan where the use of ST is common yet very little is being done to control its use.
