# Supplementary material for: Smokeless Tobacco Cessation Support in Dental Hospitals in Pakistan: Dentists and Dental Patients’ Perspectives on Current Practices, Support Needed, and Opportunities Available
Source: Nicotine Tob Res. 2023 Jul 25;26(1):63–71. doi: 10.1093/ntr/ntad125 (PMC10734380; doi:10.1093/ntr/ntad125)
Supplement: ntad125_suppl_Supplementary_Appendix_S4 [file ntad125_suppl_supplementary_appendix_s4.docx]

**Appendix 4: Coding tree for dentists interviews**
