# Supplementary material for: Smokeless Tobacco Cessation Support in Dental Hospitals in Pakistan: Dentists and Dental Patients’ Perspectives on Current Practices, Support Needed, and Opportunities Available
Source: Nicotine Tob Res. 2023 Jul 25;26(1):63–71. doi: 10.1093/ntr/ntad125 (PMC10734380; doi:10.1093/ntr/ntad125)
Supplement: Supplementary file 5 [file ntad125_suppl_supplementary_appendix_s5.docx]

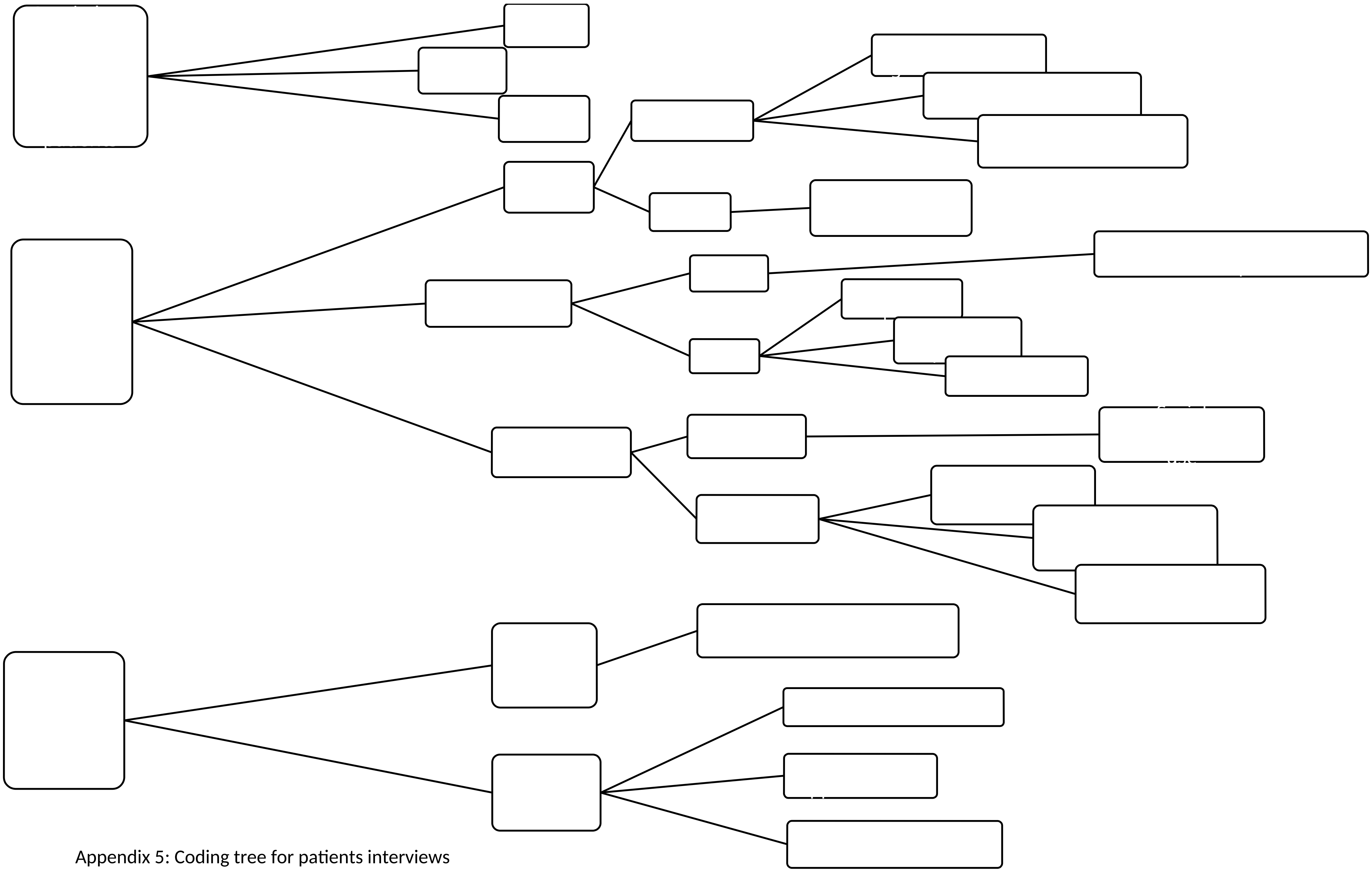

Appendix 5: Coding tree for patients interviews
